# Real Life Changes in Physical Activity Due to Intragastric Balloon Therapy and Their Relationship to Improving Cognitive Functions: Preliminary Findings

**DOI:** 10.1007/s11695-020-04440-4

**Published:** 2020-02-04

**Authors:** Agata P. Gaździńska, Aleksandra Mojkowska, Michał Janewicz, Marek Binder, Piotr Zieliński, Stefan P. Gazdzinski

**Affiliations:** 1 Department of Nutrition and Obesity, Military Institute of Aviation Medicine, 54/56 Krasinskiego str, 01-755 Warsaw, Poland; 2 Department of Surgery, Military Institute of Aviation Medicine, 54/56 Krasinskiego str, 01-755 Warsaw, Poland; 3 Department of General, Gastroenterological and Oncological Surgery Collegium Medicum, Nicolaus Copernicus University, St. Joseph’s St. 53-59, 87-100 Torun, Poland; 4 SWPS University of Social Sciences and Humanities, Chodakowska 19/31, 03-815 Warsaw, Poland; 5 Department of Psychophysiology, Jagiellonian University, Kraków, Poland; 6 Department of Psychology, Military Institute of Aviation Medicine, 54/56 Krasinskiego str, 01-755 Warsaw, Poland; 7 Department of Neurosciences, Military Institute of Aviation Medicine, 54/56 Krasinskiego str, 01-755 Warsaw, Poland

**Keywords:** Physical activity, Intragastric balloon, Endoscopic bariatric therapy, Weight reduction, Cognitive functions, Obesity

## Abstract

**Background:**

We evaluated if the intragastric balloon (IGB) treatment leads to the increase in physical activity (PA) and whether they are related to cognitive improvements.

**Methods:**

Fourteen morbidly obese patients (151 ± 24 kg, BMI = 51.8 ± 6.5, 107 ± 26% excess weight, 43.3 ± 10.6 years) underwent 6-day-long, uninterrupted evaluations of PA 1 month before IGB insertion and 1 month after its removal.

**Results:**

Active energy expenditure and physical activity duration increased by more than 80% (*p* < 0.001) whereas the number of steps per day by 20% (*p* = 0.016). There was a pattern of relationships between cognitive improvements and increases in PA (*p* < 0.05). In particular, working memory improvements correlated with the increase in time spent on light physical activities (*r* = 0.673, *p* = 0.004).

**Conclusion:**

The relationships suggest that an increase in physical activity mediates cognitive improvements in bariatric patients.

## Introduction

Endoscopic insertion of the intragastric balloon (IGB) is a minimally invasive procedure leading to premature satiation, prolonged satiety, and influences the hormone levels regulating energy balance [1–7] The weight reductions induced by IGB are retained by a relatively low number of patients over longer periods after its removal [1, 8–10]. As physical activity (PA) plays an important role in promoting long-term weight maintenance [11], we have evaluated changes in PA accompanying IGB treatment.

Weight loss is associated with improvement in cognitive functions among overweight, obese [12], and morbidly obese patients [13–15]. Previously, we have reported improvements in verbal short-term memory, visual short-term memory, and sustained and divided attention among patients treated with IGB [16]. However, it is well established that increases in PA lead to cognitive improvements, especially in executive functions and short-term memory [17, 18]. Such effects were also described in obese, but not bariatric, populations [19]. We hypothesized that an increase in PA following IGB treatment will be associated with improvements in cognitive performance on tests of visual short-term memory, sustained and divided attention, and working memory.

## Materials and Methods

### Study Participants

**Fig. 1:**
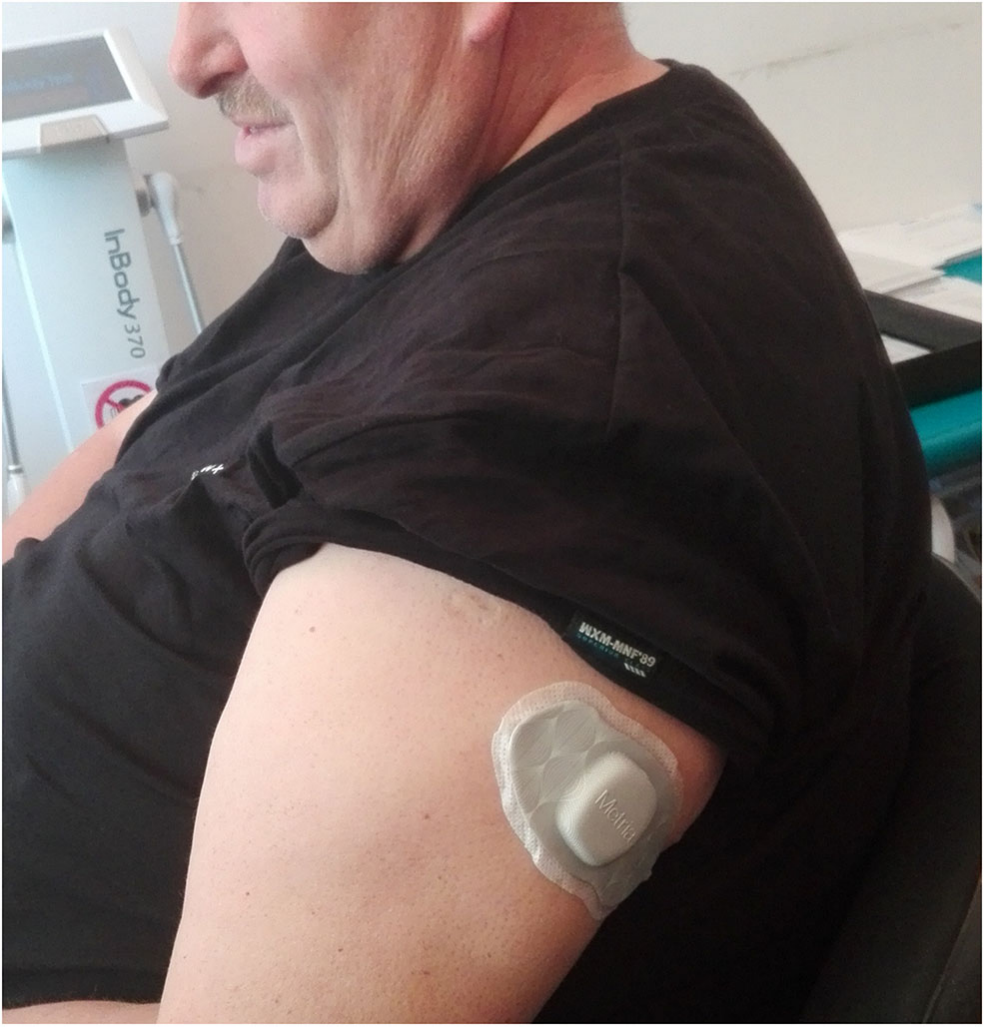
Metria IH1 device that continuously measures physical activity over 1 week

Thirty patients were enrolled in a neuroimaging study evaluating functional and structural brain changes accompanying IGB-induced weight loss [16, 20–22]. They were consecutive patients qualified for 650-ml saline-filled IGB treatment lasting 6 months (in accordance with the manufacturer’s recommendations) between April 2015 and December 2016, who did not have contraindications for magnetic resonance. The study was performed twice: 1 month before IGB insertion and 1 month after its removal. This timing was adjusted to minimize the adverse effects of the IGB procedures on the measures of interest. Each study included MRI in the morning hours (results described in [20]), followed by cognitive testing. Before discharging, devices evaluating physical activity were attached to the patients’ forearms at every visit (Fig. 1). Although these devices had been earlier tested on soldiers during military training, they did not adhere to the skin of the patients and detached. This problem was later fixed by the manufacturer; therefore, we only have data on 14 morbidly obese patients (43.3 ± 10.6 years, 7 females, 8 with comorbid type 2 diabetes, 151 ± 24 kg, BMI = 51.8 ± 6.5, %EW = 107 ± 26% excess body weight).

One patient had a gastric ulcer hemorrhage caused by taking aspirin against medical advice. This led to premature IGB removal 1 month prior to the scheduled time [23]. However, his PA measures were within the range of results of other patients taking part in the study. Detailed information on the participants is provided in Table 1.

**Table 1:** Demographics and medical information. OSA = obstructive sleep apnea

Parameter	
Age [years]	43.3 ± 10.6
Type 2 diabetes mellitus [*n*]	8
Females [*n*]	7
Weight [kg]	151 ± 24
BMI [kg/m^2^]	51.8 ± 6.5
%EW [%]	107 ± 26
Hypertension [*n*]	10
Dyslipidemia [*n*]	4
Fatty liver [*n*]	7
Cigarette smokers [*n*]	3
Gastritis [*n*]	8
*H. pylori* positive [*n*]	6
OSA [*n*]	2
Diabetic microangiopathy [*n*]	1

Ten patients met criteria for hypertension when their blood pressure was measured, four had dyslipidemia, seven had a fatty liver diagnosis, and three were cigarette smokers. Eight patients had a history of gastritis, six were *Helicobacter pylori* positive, one had diabetic polyneuropathy and diabetic retinopathy, and two had obstructive sleep apnea. The detailed demographic and medical information on the subjects 1 month prior to entry into the treatment is provided in Table 1.

All participants gave written informed consent to all procedures prior to the study. All procedures had been approved by the Institutional Review Board of the (BLINDED) and have been performed in accordance with the ethical standards as laid down in the 1964 Declaration of Helsinki and its later amendments or comparable ethical standards.

### Measuring Methodology

PA was assessed using Metria IH1 devices (Vandrico, USA, Fig. 1.) that automatically evaluate physical activity duration and active energy expenditure (AEE) for at least 5 days (up to 1 week). This device provided continuous monitoring of the parameters of interest. Any detachment from the arm by the patient would be recorded and such data would be discarded. AEE was defined as tasks at more than 1.5 METs (1 MET = energy expenditure of a person sitting quietly). Average parameters describing physical activity (see Table 2) were calculated only over the days when the device was attached to the skin of the patient for the entire 24 h. The device was attached to the skin in the vast majority of the patients for 6 days.

**Table 2:** Measures of physical activity before and after IGB insertion

	Month before insertion	Month after insertion
Total EE [kcal]	3630 ± 700*	3370 ± 710
Active EE [kcal]	560 ± 370*	1040 ± 550
PA Duration [min]	102 ± 70*	212 ± 96
Steps [n]	5520 ± 1880*	6600 ± 1820
Lying down [min]	470 ± 150	480 ± 130
Sleep [min]	340 ± 110	370 ± 120
Mean MET	1.01 ± 0.08*	1.11 ± 0.08
Sedentary [min]	1290 ± 70	1220 ± 100
Light [min]	130 ± 60*	180 ± 80
Moderate [min]	16.6 ± 16.6*	30.9 ± 30.2
Vigorous [min]	0.0 ± 0.0	3.6 ± 12.9

The grades of physical activity were defined as follow: sedentary (< 1.5METs), light (1.5–3.0 METs), moderate (3.0–6.0 METs), and vigorous (6.0–9.0 METs). Two participants had activity at vigorous level after IGB removal. No participant had ever activity at very vigorous level (> 9.0 METs). Total EE = total energy expenditure (includes resting metabolic rate). PA = physical activity

*Indicates statistically significant change

The patients also underwent tests of visual short-term memory (Benton Visual Retention Test, [24]), visual search and sustained and divided attention (Color Trail Test, CTT-1, CTT-2, (BLINDED) normalization [25]), auditory attention, and verbal working memory (Digit Span from WAIS-R, (BLINDED) version revised and renormalized in 2004). Parallel versions of the tests were utilized in random order.

### Statistical Analyses

The changes in individual parameters were evaluated using paired *t* tests. Percent changes in the measures were provided. Relationships between measures and their changes were evaluated with the Pearson product-moment correlation coefficient; the results were visually checked for outliers. All correlational tests were conducted with R version 3.4.3 (R Core Team, 2017 R: A language and environment for statistical computing. R Foundation for Statistical Computing, Vienna, Austria. URL https://www.R-project.org/). Given the small sample size, we considered correlations at significance level of 0.01 (or less) statistically significant.

## Results

### Changes in Physical Activity

The detailed information on parameters describing PA is provided in Table 2. Over the entire treatment (between 1 month IGB before insertion and 1 month past its removal), there was a 490 ± 440 kcal (87%) increase in active energy expenditure (*p* = 0.001), 110 ± 80 min (109%) increase in physical activity duration (*p* < 0.001), 1100 ± 1700 (20%) increase in number of steps per day (*p* = 0.016), 0.10 ± 0.14 (10.2%) increase in average MET (*p* = 0.008), 46 ± 71 min (35%) increase in light activities (*p* = 0.015), and 14 ± 19 min (86%) increase in moderate activities (*p* = 0.007), accompanied by 65 ± 79 min (5.1%) decrease in time spent on sedentary activities (*p* = 0.005). These changes were overlaid on a 7% (*p* = 0.03) decrease in total energy expenditure, driven mainly by the decrease in resting metabolic rate that is generally observed [22].

### Correlations

**Fig. 2:**
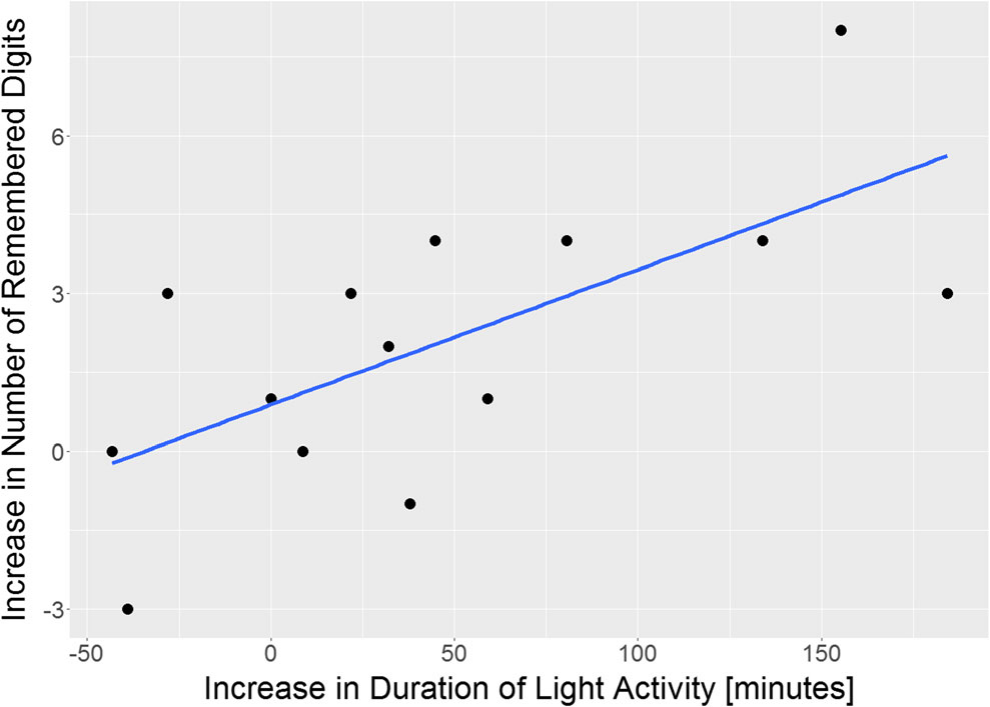
Improvements in working memory scores as a function of lengthening of light physical activity. Please not that negative improvements represent cases, where the patients remembered less items after IGB removal that before

Over the entire treatment, between insertion and removal of IGB, improvements in working memory were related to increases in the duration of light physical activity (*r* = 0.673, *p* = 0.004, Fig. 2) and tended to correlate with the increase in the number of steps (*r* = 0.514, *p* = 0.03). These improvements also tended to be related to the shortening of sedentary activities (*r* = − 0.591, *p* = 0.03). A decrease in the total number of errors on the Benton test tended to be related to an increase in AEE (*r* = − 0.600, *p* = 0.012) and increases in MET (*r* = − 0.476, *p* = 0.043). Decreases in execution time on Color Trail Test (CTT-1) tended to be related to decreases in body mass, BMI, and %EW (*r* > 0.52, *p* < 0.042). Interestingly, no changes in PA correlated with changes in body mass, BMI, and %EW (|*r*| < 0.38, *p* > 0.26).

## Discussion

We have observed weight reduction in a group of fourteen morbidly obese patients treated with an intragastric balloon between 1 month prior to IGB insertion and 1-month post-IGB removal. Substantial increases in physical activity were recorded. In particular, increases in energy expenditure and PA duration were accompanied by a reduction in time spent on sedentary activities. These changes were related to improvements in working memory.

The observed weight reduction in our study was comparable to previous reports [3, 4, 6]. Slightly larger weight reduction in our study (about 23 kg vs. 17–18 kg) [3, 4] or 11.7 kg [26] could have resulted from the longer, 8-month interval in our study, as compared to 6 months in studies measuring differences between weight on the day of IGB insertion and IGB removal. Conversely, when the interval was longer than in our study, some patients started to regain weight; thus, the reported reductions were also slightly lower than in our study (see [6]).

The patients increased the intensity of their physical activity, somewhat in contrast to earlier studies that did not demonstrate any significant increases in PA following bariatric surgery [27, 28]. In contrast to the earlier studies, our participants had continuous PA measurement over 6 days, whereas, e.g., in other studies, the measurement was carried out only several hours a day, e.g., more than 10 h a day for 4 days [28]. Self-assessment questionnaires were not used in our study, as patients tend to overestimate their physical activity changes [27, 28]. The large increases in PA in our cohort likely reflect the very low level of PA at the baseline. Despite the improvements, the WHO-recommended level of 10,000 steps/day was not reached. Long-term changes in PA after IGB removal and their relationship to bodyweight need to be evaluated, given their potential influence on long-term weight maintenance in patients treated with IGB. Therefore, PA changes and their effects on long-term weight reduction and weight maintenance should be evaluated. Furthermore, the use of IGB registered for 12 months use should be evaluated in future studies.

## Conclusions

To our knowledge, this is the first study to evaluate correlations between increases in physical activity and cognitive improvements in bariatric populations. Intragastric balloon therapy is associated with substantial increases in PA. They correlated with improvements of working memory and showed tendencies with improvements on other tests. Further studies are needed to evaluate the long-term effects of PA in the long-term maintenance of reduced body weight.
